# Sensors and Functionalities of Non-Invasive Wrist-Wearable Devices: A Review

**DOI:** 10.3390/s18061714

**Published:** 2018-05-25

**Authors:** Aida Kamišalić, Iztok Fister, Muhamed Turkanović, Sašo Karakatič

**Affiliations:** Institute of Informatics, Faculty of Electrical Engineering and Computer Science, University of Maribor, Koroška Cesta 46, 2000 Maribor, Slovenia; iztok.fister1@um.si (I.F.J.); muhamed.turkanovic@um.si (M.T.); saso.karakatic@um.si (S.K.)

**Keywords:** wrist-wearable device, non-invasive, sensor, intelligent analysis, visualization, taxonomy

## Abstract

Wearable devices have recently received considerable interest due to their great promise for a plethora of applications. Increased research efforts are oriented towards a non-invasive monitoring of human health as well as activity parameters. A wide range of wearable sensors are being developed for real-time non-invasive monitoring. This paper provides a comprehensive review of sensors used in wrist-wearable devices, methods used for the visualization of parameters measured as well as methods used for intelligent analysis of data obtained from wrist-wearable devices. In line with this, the main features of commercial wrist-wearable devices are presented. As a result of this review, a taxonomy of sensors, functionalities, and methods used in non-invasive wrist-wearable devices was assembled.

## 1. Introduction

Non-invasive devices are used for the continuous monitoring of health, exercise activity, assessing performance and other monitoring activities. With real-time information, those devices allow individuals to change their lifestyle, optimize exercises or training, prevent hazards, and optimize sleep patterns, among other use cases. Different sensors are combined and incorporated into wrist-wearable devices to be used in a plethora of applications. Devices, when commercially available, are known as Commercial-Off-The-Shelf (COTS) wearables. They are used in a variety of domains, such as medicine, education, sports, and construction.

The application of wrist-wearable devices are possible in different domains:

- medicine—to monitor a patient’s health (as well as activity and environmental parameters) in order to prevent injuries and allow for the early detection of illnesses and/or disorders as well as early interventions, offer preventive actions to avoid the deterioration of a health condition [1,2,3,4,5,6,7,8,9,10,11,12,13,14,15,16,17,18,19,20,21,22,23,24,25,26,27,28,29,30,31,32];
- risky professions (such as construction, firefighting, aviation, and military)—to monitor a person’s vital health functions (as well as activity and environmental parameters) to prevent potential risky situations such as accidents and injuries and offer corrective or preventive actions to be undertaken to avoid further deterioration or risk [10,33,34,35,36,37,38];
- education—monitoring stress levels and health conditions to offer personalized learning plans, time management recommendations, the scheduling and development of classroom activities, and establishing students’ working groups [2,36,39,40,41,42];
- office environment and industry—to monitor employee’s health parameters, especially monitoring stress levels and to prevent the potential deterioration of health conditions caused by occupational stress [17,29,36,43,44]; and
- sports (recreative or professional) and lifestyle—monitoring health parameters and training activities (as well as environmental parameters) to prevent potential injuries, to reach optimal fitness, help change lifestyle, assess sleep quality, and so on [10,12,15,16,17,27,28,29,45,46,47,48,49,50,51,52,53,54,55,56,57,58,59,60,61,62,63,64,65].

Wrist-wearable devices using non-invasive measurements can contain: sensors which produce raw data of measurements, computers (integrated into a wearable device or external) for intelligent analysis of raw data obtained from sensors and monitors that visualize data obtained from sensors or results of the intelligent analysis. There are many commercial devices available today but there are also different studies that focus their research on sensors and intelligent analysis of data obtained from those sensors integrated into prototypes which are not available as commercial devices.

Here, we provide a comprehensive review of sensors used in wrist-wearable devices, methods used for the intelligent analysis of data obtained from wrist-wearable devices and methods used for the visualization of parameters measured. Commercial wrist-wearable devices were analyzed and key features examined. The subsequent review resulted in a taxonomy of sensors, functionalities, and methods used in non-invasive wrist-wearable devices while also identifying existing issues (such as the accuracy of sensors, homogenization of data, lack of on-device intelligent analyses, and a shortage of intelligent analysis visualization techniques) which will be discussed in detail in the following sections.

The structure of the paper is as follows. Section 2 presents research methodology used in the review, research questions and taxonomy. In Section 3, the main features of commercial wrist-wearable monitoring devices are presented. A detailed analysis of sensors used in wrist-wearable devices is provided in Section 4. Section 5 and Section 6 provide an analysis of the methods used for data visualization and intelligent data analysis, respectively. A discussion can be found in Section 7. Finally, the conclusions and future trends are presented in Section 8.

## 2. Research Methodology

This research was conducted by reviewing the present state of knowledge of wrist-wearable sensors and devices.

While designing the research, we formed two research questions, which are addressed and answered in the paper. The research questions are as follows:

**Research** **Question** **1**  (RQ1)**.** *What are the core elements for defining the taxonomy of wrist-wearable devices?*

**Sub-Question** **1** **** (SUB1-RQ1)**.** *Which sensors are used for measuring physiological, activity and environmental parameters with wrist-wearable devices?*

**Sub-Question** **2** **** (SUB2-RQ1)**.** *Which methods are used for the intelligent analysis of data obtained from wrist-wearable devices?*

**Sub-Question** **3** **** (SUB3-RQ1)**.** *Which methods are used for data visualization on wrist-wearable devices?*

**Research** **Question** **2**  (RQ2)**.** *How are research findings applied to the current commercial devices?*

Based on the research questions, the review was undertaken to address five specific goals:

- To review sensors used in wrist-wearable devices. The literature review revealed three divisions: physiological sensing, environmental sensing, and activity sensing.
- To review methods used for the intelligent analysis of data obtained from wrist-wearable devices.
- To review methods used for the visualization of parameters measured with wrist-wearable devices.
- To review popular commercial wrist-wearable devices and provide a feature comparison for those devices.
- To synthesize a taxonomy of sensors, functionalities, and methods used in wrist-wearable devices.

To reach these goals, first a comprehensive literature review was performed. Publications were retrieved using the computerized search of the following citation indexing databases and search engines of publishers: Sage, Emerald, Google Scholar, MDPI, Science Direct, Web of Science, Wiley, IEEE Xplore and SpringerLink, to find relevant studies published from the last five years. Furthermore, an online search was carried out to identify the leading manufacturers of wrist-wearable devices. Manufacturers’ specifications documents (white papers and manuals) and published research work were reviewed, and the obtained information was evaluated.

The research has several limitations (inclusion criteria):

- limited to wrist-wearable devices;
- limited to sensors which are used as an integral part of a prototype or commercial devices; and
- limited to devices which are independent of devices for data collection and intelligent data analysis.

The search string used to retrieve publications was: (wrist OR wrist-worn OR wristband OR wrist-wearable OR smartwatch OR smartband) AND (wearable OR device OR sensor OR wearable device OR gadget OR iot) AND (track* OR mining OR acquisition OR monitor*). After removing duplicates, the titles and abstracts of the remaining papers were reviewed, and those that did not encompass the inclusion criteria were discarded. The search results showed that the first two limitations regarding set inclusion criteria can be taken into account, but the third limitation left our search with very few results. Therefore, we adjusted our search to also review and evaluate devices that are only data collectors (i.e., intelligence is present only on the web or on another device). The bibliographies of all relevant articles and review papers were also reviewed by hand. The remaining papers were read in-depth.

### Taxonomy

**Figure 1:**
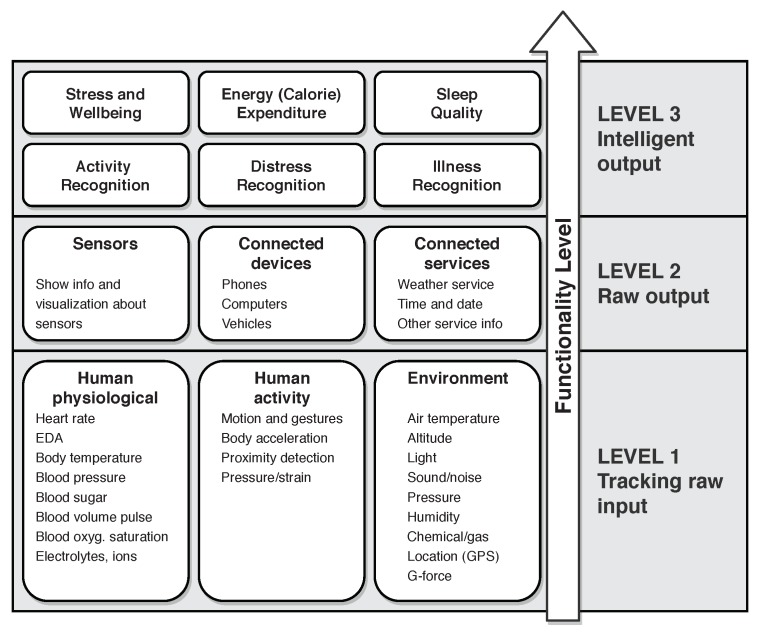
The taxonomy of functionalities of wrist-wearable devices with three levels. Our paper focuses on wrist-wearable devices with Level 3 functionalities of intelligent output.

Through our literature review of smart wrist-wearable devices, we found a lack of clear definition of the taxonomy of those devices with regard to functionality. Thus, we present our taxonomy of wrist-wearable devices based on their functionalities which are depicted in Figure 1. There are three functionality levels in the taxonomy of wearables, where a device on a level greater than the first one can (but not necessarily) include some functionalities from lower levels. The focus of this paper is on wrist-wearable devices at the Level 3.

Level 1 of the taxonomy consists of pure raw input trackers. All of the basic wearable trackers are categorized here: heart rate, blood pressure, temperature, humidity, light, sound, motion, acceleration, and others. The basic distinction between Level 1 functionality wearables and higher functionality level wearables is that devices in this level only track and/or log the raw inputs from human physiology, activity or environment and do not share this information directly with the user who wears them.

Level 2 functionality wearables include some raw input monitoring and complement this with some raw output indicators. These outputs can be in the form of a graphical interface such as LCD, e-ink or another type of display, sound, vibration, light with monochrome or multiple colors and patterns of flashes, mechanical indicators and other types of visual, sound or feel indicators. The typical representative of Level 2 functionality wearable is a sports tracker, which monitors some physiological inputs and then displays this value back to the user. Some wearables can also show raw inputs that are gathered by a remote device (be it a remote weather station or a separate heart rate tracker). These types of devices are also categorized in the Level 2 even though they do not include a raw input monitoring system.

The final level of wearable functionality taxonomy is Level 3, which includes wearables that output some intelligent information to the users. In contrast to Level 2 devices that show only raw values, Level 3 wearables process these inputs in the number of different ways to show some new information derived from the inputs. These devices come in the form of activity trackers, smart watches, safety wearables and such. Their key characteristic is that they implement some transformation of raw inputs, which can be done through well known mathematical formulations, rule sets, statistics or even machine learning models. For example, energy expenditure cannot be directly measured, so it must be approximated with the use of predefined formulas with inputs, such as weight, height, heart rate, the age of the user, travelled distance and others.

The taxonomy does not force the calculation of the intelligent output to be on the wearable device itself—it can be done remotely in the cloud or other connected devices (smartphone or computer). A Level 3 device does not even need to track the raw inputs itself—this can be done in conjunction with other sensors. In this sense, a Level 3 device can just be an indicator that either transforms the raw inputs to new knowledge or gathers transformer raw inputs to knowledge remotely and shows this to the user.

## 3. Commercial Devices

The objective of this section is to introduce readers to some commercial solutions among wrist-wearable monitoring devices. We conducted an extensive market research (see Table 1 for information about market share) about commercial products that can be bought on the current market as well as to find an answer to the question: “Which devices are currently TOP devices?” Due to the popularity of wrist-wearable monitoring as well as tracking devices, the market is full of solutions that can be used for various domains like sport and fitness, extreme sport (e.g., diving), medicine, support for office work and many more.

However, after an extensive analysis of products and their white papers, we found that we cannot rank commercial solutions on one scale. Each device is specific and intended for a particular purpose. In line with this, some people even do not look for the functionalities of a device; instead, their goal is to have a good-looking device. Therefore, they place a special attention on the design of the device.

In parallel, we also reviewed some online rankings of commercial devices that were compiled by commercial media houses and blogs. Interestingly, these sources showed significant discrepancies between rankings. Some sources may also be paid for advertisements or trying to achieve a higher ranking for a particular device.

According to the above-mentioned arguments, we decided that mentioning any of the commercial devices might be very biased. Thus, we have only presented core elements that were found from either the device description or white-paper. When reviewing devices from the aforementioned source, we concentrated on three core elements:

- key features or main functionalities that are useful for end-users;
- sensors that are integrated within the device; and
- IT support or the possibilities of connecting devices with smartphones or other web applications.

All core elements are presented in Table 2.

Regarding the comprehensive study, we can conclude that modern devices allow end-users very complex functionalities, as for example sleep tracking, stroke detection, detection of exercise and much more [67,68]. In line with this, most modern devices provide special attention to the continuous monitoring of heart rate, which leads to numerous analyses [69,70]. Various predictions of diseases or heart problems are some examples that can enrich the user’s experience with the device. Additionally, the producers of devices also provide abundant IT support in the realm of smartphone support or supporting web applications, where users can analyze measurements that were produced by devices.

## 4. Sensors

Wearable devices use sensors to collect raw data from measurements which are stored and used for the continuous monitoring of health, exercise activity, assessing performance, and others. In addition, using a different kind of sensor is currently as modular as a plug-and-play option in the popular Arduino or Raspberry Pi computer hardware, where sensors (e.g., temperature, sound, light, and potentiometers) can directly be put into the boards [71]. Furthermore, the list of possible distinct sensor types is rapidly growing, enabling users to monitor a range of concerns including air quality, barometric pressure, carbon monoxide, capacitance, gas leaks, humidity, hydrogen, etc.

We focused on reviewing sensors used in wrist-wearable devices for non-invasive monitoring of human physiology, activity or environmental parameters. We also focused on covering sensors which could be used as part of a wrist-wearable device, while currently only marketed as part of a specific non-wrist-wearable device.

In this section, we present in detail the three types of sensor technologies, based on their focus, i.e., physiological, environmental and activity sensors. Each group is represented with a list of the sensor’s representative, aim, key functionalities, and sensor-related open issues as well as potential research directions. Table 3 summarizes the results obtained during the review, where specific parameters are related to the sensors used for their measurements.

### 4.1. Physiological Sensing

This section presents an overview of sensors used to monitor physiological parameters. The wrist can be used to obtain a vast amount of physiological signals such as temperature, heart rate, oxygen saturation or bioelectronics [13].

#### 4.1.1. Heart Rate

Heart rate (HR) or pulse is the frequency of cardiac cycles, and is expressed as beats per minute [13]. The most popular technique for HR measurement is photoplethysmography (PPG). It is used to detect volumetric changes in blood in peripheral circulation. On the wrist, the pulse signal can be obtained from the plethysmogram by using optical and pressure sensors. In the optical sensing method (i.e., photoplethysmography), PPG detects blood flow rates by capturing the light intensity reflected from skin based on LEDs and photodetectors [34]. A light source is used to illuminate the arteries, while a photo-detector collects light reflected from, or transmitted through, the tissue [1]. The transmitted or reflected light intensities through and from the skin while tissue change depends on the blood volume. The signal obtained using a photodiode [13] represents the pulsatile blood volume changes of peripheral microvasculature, induced by pressure pulse within each cardiac cycle [1]. Transmitted light can be obtained in a relatively good signal, but the measurement site is limited to those parts of the body where the signal can be quickly detected. This is not an option for a wrist-wearable device. On the other hand, a reflective signal can be used on a variety of measurement sites, including the wrist. Nevertheless, it has another limitation since it is affected by motion artefacts, pressure disturbances and skin pigmentation. The accuracy of the HR obtained with PPG sensing from a wrist can be improved using signal processing techniques [34,72].

- Fallet et al. [24] presented a normalized least-mean-squares algorithm that was used to debilitate motion artifacts and to reconstruct multiple PPG signals from different combinations of damaged PPG waves and data obtained from the accelerometer. To track the HR of the reconstructed PPG waves, an adaptive band-pass filter was used.
- Nowak et al. [73] presented methods to track HR from PPG signals. The developed methods are robust against motion artifacts. They used spectral subtraction or non-negative matrix factorization (NMF) for signal enhancement and motion artifacts removal, and online Viterbi decoding or particle filtering.
- Shilin et al. [74] proposed the MICROST framework using a mixed approach to estimate HR. It consists of acceleration classification, first-frame processing, and heuristic tracking.
- Ishikawa et al. developed a wristband-type PPG heart rate sensor which is capable of overcoming motion artifacts in daily activity and detecting heart rate variability [29]. They managed to significantly cancel motion artifacts caused by arm, finger and wrist movements.
- Noise reduced pulse signals based on peak detection and auto-correlation methods were used to instantaneously detect heart rate with high noise resistance. Fukushima et al. estimated HR on running persons using the difference between the power spectra of PPG and acceleration [75].
- Lee et al. developed frequency-multiplexed sensor array using complementary split-ring resonators [76]. Those can spatially resolve the positional changes of the wrist relative to the location of the sensors on the bracelets. Proposed method detects the perturbation of the electrical field radiated from the source resonator caused by the mechanical movement of the artery. The detected perturbation is used to verify the biological information used for the signal modulation. The bracelet combines position detection and pulse measurement to obtain a more accurate measurement of the pulse.
- FitBeat [63] is a lightweight system implemented as a wrist-wearable device that enables accurate heart rate monitoring during intensive exercises. It integrates contact and motion sensing as well as simple spectral analysis algorithms to suppress various error sources. Augmenting standard filter and spectral analysis tool enables significantly reducing computational overhead.
- Mohapatra et al. [77] proposed a sensor module design addressing anomalies due to the optical properties of skin. Their 590 nm (yellow-orange) wavelength based optical system is developed to maximize the signal quality acquired.

HR can be also derived from measured Blood Volume Pulse [44]. In addition, multi-channel WPPG sensor signals and acceleration signals can be used to measure HR [78]. Infrared technology can be used to monitor and calculate heart rate (HR). Infrared wireless conveys data through infrared radiation. According to [33] commercially available infrared sensors are used in conjunction with Bluetooth technology for connectivity. In addition, an ultrasensitive pulse driven sensor made using tellurium dioxide triangular microwires is used to obtain pulse measurements [79].

#### 4.1.2. Body Temperature

Body temperature varies depending on the measurement site. It can be estimated from the skin temperature, which can be measured with infrared thermopile [44], thermistors, thermoelectic effects or via optical means [13]. Thermistor configuration is mostly used in wearable sensors, whereas the resistance of the thermistor varies depending on the temperature [13]. Thermistors show a strain dependence. The challenging issue remains separating strain effects from temperature effects in thermistors. Another important issue is to accurately estimate body temperature. Wrist-wearable devices measure skin temperature, which is a few °C less than the body temperature. In addition, skin temperature shows oscillations depending on environmental temperature, whereas body temperature varies less than 1°C throughout the day [13]. Another challenge rests in the evaporation of sweat and in the low thermal contact between skin and the sensor, which results in a reduced relationship between skin and body temperature. There are different studies demonstrating the possibility of relating the two. In [32] the authors present skin temperature wrist measurement with added domain specific knowledge to obtain the estimated body temperature. Skin temperature together with other measurements are used to measure stress and detect emotions. It has been shown that skin temperature is negatively correlated with stress (skin temperature increases when stress levels decrease) [2,43]. Sim et al. [36] presented a wrist band that monitors skin temperature from the wrist and estimates subjective thermal sensation using the monitored wrist skin temperature.

#### 4.1.3. Blood Pressure

Blood pressure (BP) represents the pressure of the blood against the inner walls of the blood vessels due to blood circulation [2,43]. Systolic and diastolic values are used to analyze the BP. A pressure sensitive transistor is used for blood pressure wave measurement. These pressure sensors are in the form of capacitors, which use a compressible dielectric to induce a change in capacitance or a piezoelectric to induce a voltage across the device. An external instrument or a monolithically integrated device on a thin film transistor can be used to amplify the signal from the capacitive elements [13]. In addition, piezoelectric materials are used for creating strain-sensitive devices. When a piezoelectric material is strained, a voltage signal is induced across the device. Detailed information on piezoelectric materials is provided in Section 4.2.4. The system of a pulse pressure sensor is important as the sensor itself. In these systems, the amplification is necessary since the result of the background pressure is substantially larger than the pulse pressure. The amplification is provided through monolithic integration of an amplifier next to the sensor [13]. In addition, PPG sensing is used for BP measurement [1]. In the pressure sensing method, systolic peaks (the light absorption peaks due to a high volume of blood) are detected using capacitive pressure sensor for radial or the carotid artery [1,13]. Lee et al. [11] proposed an algorithm for estimating the BP. It is based on an analysis of the non-pressurized pulse wave obtained from the wrist-wearable pulsimeter with a Hall device. The developed device detects minute changes in the magnetic field of a permanent magnet. Hsu et al. [55] presented an approach to blood pressure measurement using two silicone-coated microelectromechanical (MEMS) pressure sensors in order to non-invasively detect blood pressure waveforms through skin contact. Sensors are placed at two points of the body to measure BP waveforms simultaneously. The local pulse wave velocity (PWV) can be determined by obtaining the delay time between two waveforms.

Even though there is a plethora of research demonstrating being capable to measure the pulse pressure, there are no many that were able to demonstrate the correlation between the measured signals and blood pressure. Some of those that did manage to determine blood pressure (mentioned in the paper by Khan et al. [13]) found non-linear relationships between systolic/diastolic BPs, and sensor output. We can conclude that there is still a lot to be done considering the reliability of these sensors.

#### 4.1.4. Blood Oxygen Saturation

Oxygen saturation or oxygenation is the concentration of oxyhemoglobin in the blood divided by the sum of the concentration of oxy- and deoxy-hemoglobin in the blood [13]. Due to its non-invasive nature, adequate for measurements on the wrist [38], we only took into account peripheral oxygenation ($SpO_{2}$). It is determined by an optical measurement where two LEDs, operating at two different wavelengths, alternately shine light through, or on, the sensing location. The blood volume and the concentration of oxyhemoglobin in the blood determine the intensity of the transmitted or reflected light through, or from, the tissue and skin. A photodetector is used to collect this optical signal. Hemoglobin in the blood has a different absorptivity depending on whether it is bound to oxygen or not. This fact, as well as the wavelength of light, make optical measurements possible. The oxygenation is derived from the ratio of the photodetector signal upon excitation at each wavelength and the known molar absorptivity of oxy- and deoxy-hemoglobin at each wavelength [13]. In addition, PPG sensing can be used for $SpO_{2}$ measurements [1]. Motion artefacts and ambient light interference are the most common issues that hinder the accuracy of obtained measurements. Those issues can be overcome by placing the sensor more closely to the skin as well as enabling the sensor to flex and move with the skin. Considering these research directions, organic and textile optoelectronics can be integrated into novel oximetry sensing devices [13].

#### 4.1.5. Blood Sugar

Non-invasive electrochemical sensors for monitoring skin interstitial fluid (ISF) and sweat can be mainly divided into two types: fabric-based and epidermal-based sensors [10]. Glucose in these bio-fluids is diffused from glucose in blood vessels through the endothelium or sweat glands, reflecting the blood glucose concentration [80].

Even though sweat has emerged as a very useful bio-fluid for non-invasive glucose monitoring, there are still many challenges to overcome. A key issue for achieving reliable sweat and blood glucose correlations remains sweat sampling which should be constant and without any contamination [80]. To obtain continuous sampling, the contact between the electrode and the biofluid should be constant, which can be challenging in the case of fabric-based sensors. To overcome this hurdle, an enzyme-based amperometric glucose sensor is used in the form of a temporary tattoo adhered to the skin [81]. Here, reverse iontophoretic extraction is used to transport interstitial glucose from the skin to the sensor [13,80]. Reverse iontophoresis uses a small current applied between two electrodes to move sodium ions through the skin towards the sensing electrodes. As the sodium ions flow across the skin, a flow of interstitial skin fluid is induced, transporting the glucose contained within to the sensing area [10,13].

Furthermore, researchers are focusing their investigations on combining epidermal glucose measurements with the sensing of other physiological parameters to improve the accuracy of the obtained measurements as well as to find a correlation between other physiological parameters and glucose levels. In addition, special attention should be given to technological issues such as: resiliency, long-term stability and biocompatibility. Epidermal-based sensors are subject to mechanical deformations during body movements while fabric-based sensors are faced with deformation and degradation issues due to textile washing [10].

#### 4.1.6. Blood Volume Pulse

Blood volume is the amount of blood in blood tissue during a certain time period. Blood volume pulse (BVP) measures the amount of light reflected by the skin [2]. BVP measurements can be obtained using photoplethysmography (PPG) sensing, which optically detects changes in the blood flow volume and provides information on the pulse rate [72].

HR and HR Variability can be derived from the Blood Volume Pulse [44]. HR variability (an indicator of the dynamic and cumulative load) is used as a measure to detect cardiovascular conditions and as a primary measure for stress [2].

#### 4.1.7. Electrodermal Activity

Electrodermal activity (EDA) (i.e., galvanic skin response or skin conductance) is a measurement of the flow of electricity through the skin [2,43]. The changes arise when the skin receives specific signals from the brain [44]. They may be due to emotional activation, cognitive workload or physical exertion. EDA can be measured with two electrodes placed next to each other on the skin surface with a weak electrical current applied between them [43]. EDA sensor can capture the electrical change measuring a nervous system reaction to stress, engagement, excitement, etc. Skin conductance is increased when a person is under stress due to an increase in moisture on the surface of the skin. When the person becomes less stressed, the skin conductance is reduced [2]. It is widely used in stress and emotion detection [43].

#### 4.1.8. Electrolytes and Ions

Electrolyte (e.g., $H^{+}$, $Na^{+}$, $K^{+}$, and $Na^{+}$) imbalance can cause several health problems, therefore monitoring the level of these in human fluids can enable the early prevention of some diseases (e.g., hyperkalemia and cystic fibrosis) [4]. The monitoring of electrolytes is feasible also through wrist-wearable devices using ion sensing as the analyte for wearable electrochemical sensors. There are three types of analytic methods which are classified based on the electrochemical ion sensor, potentiometric, voltammetric and amperometric sensor, of which the first is the most widely used [4]. The potentiometric sensor works with two electrodes, one being the working electrode and the other the reference electrode. The latter’s potential is determined by the fixed ion of interest, whereby the other is determined by the environment, therefore the difference can be deduced and related to the concentration of the dissolved ion. In contrast, the amperometric sensor senses the current flow in the cell level within an applied potential, while a voltammetric sensor detects the potential difference across an electrochemical cell [4].

### 4.2. Activity Sensing

This section presents an overview of sensors used for monitoring activity parameters. The wrist can be used to obtain different activity signals such as motion, gestures, rotation, and acceleration.

#### 4.2.1. Motion and Gestures

An accelerometer is the most commonly used sensor for measuring the motion of the human body. It measures acceleration in one or more axes [82]. The acceleration can be determined by duty cycles which measure the length of the positive pulse width and period. The outputs are digital signals whose duty cycles are proportional to acceleration [32]. Triaxial accelerometers (which measure vibration in three perpendicular axes) and magnetometers (measuring the strength and direction of magnetic fields) can be used to detect different human arm and body movements [82], while RGB-D sensors, in combination with triaxial accelerometers, can be used to detect 20 different human activities [23].

Gyroscope sensors are used to determine the rotation of different parts of the body. They monitor activity by measuring body rotation and angular velocity around one or more axes while magnetic field sensors are useful in determining orientation relative to the earth’s magnetic north [33,82]. Gyroscopes are mostly used in combination with accelerometers.

Surface electromyography (sEMG) is used to detect musculoskeletal movements and activities which are controlled via nerves emitting signals which can be measured at the skin’s surface. These sensors can be used to detect hand gestures. Piezoelectric pressure sensors are used for surface mechanomyography (sMMG) to recognize gestures [83]. Since the palm side of the wrist deflects noticeably during finger flexion gestures, the deflections can be measured using an array of piezoelectric contact sensors.

Wrist-based actigraph devices have been utilized in research settings for monitoring sleep quality [84].

Usually, the gyroscope, accelerometer and magnetometer are combined in the same device, since each sensor has its own strong sides. For example, the gyroscope reacts quickly to changes and is more reliable in the measurement of angles, while the magnetometer has poor accuracy for fast movements but with no deviation over time [33].

#### 4.2.2. Body Acceleration

Malhi et al. [32] used an accelerometer as an impact sensor for measuring accelerations up to ±2 g. The acceleration can be determined by duty cycles which measure the length of the positive pulse width and the period. The outputs are digital signals whose duty cycles are proportional to acceleration.

#### 4.2.3. Proximity Detection

There are many proximity avoidance systems developed using various technologies, such as an ultrasonic-based sensor, radio frequency sensing, magnetic field, radar, sonar and GPS to prevent contact accidents. These systems are especially used in construction environments to prevent accidents caused by collisions with the equipment [33]. Awolusi et al. [33] argued that the most commonly used system for proximity detection is Radio Frequency Identification (RFID). It is based on the projection of radio waves and signals to transmit data and conduct wireless data retrieval and storage to identify the status of persons and objects [33]. The advantage of ultrasonic-based sensors is in their good resistance to background noise, while a disadvantage is in the possibility of erroneously responding to loud noises. Additionally, the main advantage of GPS-based systems is in its ease-of-use and high accuracy. Measurements such as position, speed and orientation are the advantage of GPS-based systems over other technologies, such as ultrasound, radio frequency and infrared sensing.

#### 4.2.4. Pressure/Strain

Strain sensors are used to track human body pressure information. Conventional strain sensors, are usually made of thin metal foils or semiconductors and can be placed on multiple body parts, thereby sensing that specific body part’s strain changes. Strain sensors can also be placed on a wrist-wearable, thereby sensing and recording wrist movement, useful for specific professions (e.g., tennis player) [85]. There are three common strain–pressure sensor types: (1) piezoelectric; (2) capacitive; and (3) FET-based type [18]. The former two are the most commonly used ones. The piezoelectric type reads electrical charges generated by external forces (i.e., pressure, strain). Such piezoelectricity is generated only when materials with noncentrosymmetric crystal structures are used (e.g., ZnO nanowires and lead zirconate titanate), thus being ultra fast [18]. These materials are however costly for manufacturing and have high lead toxicity, thus polymer-based piezoelectric materials are also used (e.g., PVDF) [18]. For flexible capacitive-type strain sensor, usually golden films are used as top and bottom electrodes, which produce flexible capacitive devices. However, nowadays there are also experiments with carbon nanotubes and silver nanowires [18].

### 4.3. Environmental Sensing

This section presents an overview of sensors used for monitoring environmental parameters. Environmental parameters can present challenges for accurate physiological sensing due to its direct or indirect effect on those. Avoiding environmental effects is almost impossible in a person’s natural environment, therefore it is desirable to have a thorough knowledge of its state, thereby being able to deduce the effect it has on physiological parameters [80].

According to McGrath et al. [46], environmental monitoring can generally be categorized into indoor and outdoor. The former typically focuses on home and/or workplace environments, thus measuring temperature, humidity, light, quality of air, noise and gasses. The later cover air pollution, water quality, traffic noise, weather among others. In this section, we present some of the environmental sensors.

#### 4.3.1. Air Temperature

The reflection of the rotational, vibrational and translational motion of physical matter represents the temperature itself. There are four common types of thermal sensors: (1) Negative Temperature Coefficient (NTC) thermistor; (2) Resistance Temperature Detector (RTD) or resistance thermometer; (3) thermocouple; and (4) semiconductor-based sensors [86]. An NTC thermistor is a highly accurate thermally sensitive resistor. RTD is based on the resistance of the RTD element with temperature and can consist of a film or a wire wrapped around ceramic or glass. Thermocouple sensors, as the name suggests, consists of two wires of different metals connected, whereby the varying voltage represents the proportional changes in temperature. Semiconductor-based sensors are based on two identical diodes with temperature-sensitive voltage and are placed on integrated circuits [86,87]. It is also noted that some capacitive and resistive read-outs have been integrated in a polyimide foil for temperature detection [33]. A part of an ISO standard is the so-called predicted mean vote (PMV), which aims to anticipate the average thermal sensation of a mass, whereby the thermal sensation is the sense of temperature between hot and cold [36]. Using the data of the air temperature in combination with skin temperature is a useful pointer towards assessing an individual’s thermal sensation [36]. Each of the mentioned thermal sensor types has its own shortcomings. NTCs work best for low temperatures and due to its exponential nature, requires linearization for its output. RTDs are commonly made from nickel and copper but these are not as stable or repeatable, thus platinum is a better choice albeit a far more expensive one. The thermocouple and semiconductor-based sensors have low accuracy, while the latter also have the slowest responsiveness [86,87].

#### 4.3.2. Altitude

Altitude is commonly determined using barometric pressure sensors (MEMS) [46]. These barometric pressure sensors are also used in embedded devices (e.g., mobile phones) for vertical position detection based on barometric altitude readings [88]. The barometric sensor has two outputs: the pressure value and the temperature, which when combined are converted into altitude value [89]. Combining these sensor measurements with GPS data improves the accuracy of the navigational systems [88]. Recording the altitude readings are desirable for sporting activities, such as climbing or diving.

The most common challenges of barometric pressure sensors are stability related and thereby accuracy, since with time they fall outside of the acceptable range of performance (i.e., installation shift, drift). Especially piezo-resistive pressure sensors drift over time [90]. One problem is also the fact that pressure sensors have to breathe under dry, humid and wet conditions, which can be a challenging task in wet environments [91]. Often changes in barometric pressure result in inaccurate pressure measurements, which could be resolved with differential-pressure measurements [90].

#### 4.3.3. Light

Sensing light intensity is quite common in current smartphones, whereby the brightness of the display is in harmony with the external light, based on the readings of the illumination sensor. These illumination sensors are typically silicon PIN-type photodiodes, photodetectors that convert light into either current or voltage [92]. These can receive wavelengths from 400 to 1100 nm [46]. Photons striking the depletion region of a diode may hit an atom, releasing and creating a free electron and a positively charged electron hole, whereby one moves towards the anode, and the other towards the cathode, creating a current [46]. There are also other types of photodetectors, such as phototransistors and light-dependant resistors [46].

The light sensor in the form of ambient ultraviolet or fluorescent light sensing in wrist-wearable devices is also present e.g., in the Fitbit Surge or Microsoft Band [41].

#### 4.3.4. Sound/Noise

Sound sensors are commonly used in several smart devices (e.g., mobile phone) or in dedicated devices such as the infant sound and movement sensor device (e.g., Angelcare). However, placing such a noise sensor and recording option on a wrist-wearable device, could be used to analyze a person’s stress level and its correlation with the environmental noise level (e.g., noise pollution in urban areas) or just by measuring the pitch level [43]. A prototype for such a wearable device has already been tested [93]. In some cases, piezoelectric pressure sensors are used to detect changes in pressure due to sound vibrations, thus filtering decibel readings [4,46].

#### 4.3.5. Atmospheric Pressure

There are several wearable pressure sensors, which detect different pressure ranges, i.e., under 10 kPa, between 10 and 100 kPa and over 100 kPa. The high pressure range (>100 kPa) covers the atmospheric pressure (e.g., also vocal cords), while others are too sensitive, thus, worn on a wrist, it can be used to detect blood pressure [4]. These pressure sensors are based on mechanisms which include piezoeletrics, piezoresistive and capacitance, which turn atmospheric signals into environmental information [4]. The most commonly used pressure sensor, the piezoelectric, is based on electrical charges occurring in specific solid materials (e.g., lead titanate) under pressure, thereby causing polarization, which is proportional to the applied pressure. These pressure sensors have a fast response time and are thus also used for sound detection, due to the sound vibrations that cause dynamic pressure. A piezoresistive pressure sensor can detect a wide range of pressures and is based on the resistance of specific materials, which in turn changes when physical pressure is applied. Capacitive pressure sensors are based on capacitors and on the thickness of dielectric materials (e.g., polyurethane), thus proportionately, when thickness reduces, capacitance increases [4]. An example of a wrist-wearable that enables the detection of atmospheric pressure is the LG Watch R [41].

#### 4.3.6. Humidity

The air quality also depends on the humidity level; therefore, many stationary sensory devices measure the environmental air on a specific scale. Nevertheless, such humidity measuring can also be applied on movable sensor devices, as the SensPod from Sensaris, which, besides humidity, has sensing capabilities including several gasses, noise and temperature [46]. Gathering the humidity level on the go can also be performed while being deployed on a wrist-wearable. Humidity sensing can be performed with fiber-based technology. More precisely, luminescent systems with fluorescent dyes, moisture-absorbing fiber-coating polyimides and reflective, thin, film-coated tin dioxide and titanium dioxide fibers [33,46].

These sensors also have some challenges, which, if not addressed, pose problems for accurate readings. First, the wide humidity and temperature ranges, which can exist in an environment, add to imprecise measurements and thus give a non-linear response.

#### 4.3.7. Chemical/Gas

Gas sensors, which are commonly placed in/on a physical location, are highly desirable in sensitive environments (e.g., mines, schools and nuclear facility), since they provide an additional security feature in the case of the presence of a hazardous gas, thus automatically alarming the affected public. Furthermore, such sensors can also be attached directly on a person (e.g., human skin) enabling dynamic gas detection, thus protecting the individual in case of hazardous gas detection. Gas sensor are commonly focused on molecules of specific gasses (e.g., CO${}_{2}$ and NO${}_{2}$) [4]. Gas detection is based on sensing mechanisms, such as resistive mechanism, piezoelectric devices, surface acoustic waves, electrochemical processes and colorimetric methods [4]. There are also capacitive read-outs which have been integrated into a polyimide foil, for gas detection [33].

Resistive gas sensors based on graphene (they can also be based on metal oxide, conducting polymers or colloidal nanocrystals) are commonly used in wearable gas sensors, because of their flexibility, surface-to-volume ratio and electric response [4]. The electrochemical-based gas sensors operate on the notion of gas molecules reacting and creating electrical current flow due to two sensing electrodes separated by an electrolyte. These types of gas sensors are promising for wearable devices due to low power requirements, high sensitivity and low cost. Another promising gas sensor type for wearable devices are colorimetric gas sensor, which produce visual signals due to their flexibility, reliability and have none of the disadvantages that come with electrical issues such as electronic circuits [4].

Due to their architectural design, wearable chemical sensors experience stability problems with lower power, since these require an active power source to bias the sensor, amplify the output and calibrate the sensor [94]. Additionally, some of the main challenges for chemical sensors are viable materials and fabrication techniques [95].

#### 4.3.8. Ultraviolet (UV) light

The ultraviolet light (UV) spectrum is known to be harmful for human skin, causing problems from wrinkles to skin cancers. Measuring UV exposure can be performed with specific light sensors, which can be placed on wristbands (My UV Patch [14]), bracelets or wristbands (Microsoft Band [41]) [96]. Such UV sensor backed smart devices can be used as a safety precaution. The UV sensors are part of the optical sensor family, whereby each of them are sensitive for a specific spectral region, i.e., ultraviolet, infrared, etc. These sensors are based on the principles of photoconductivity, whereby they measure changes in light intensity in relation to light emissions or even the changes of light beams in relation to any other interaction [97].

#### 4.3.9. Location

Location-based sensing is primarily done using GPS trackers or Inertial Measurement Unit (IMU) but are sometimes combined with other sensors such as accelerometers, gyroscopes, magnetometers and pressure sensors [37]. Such readings are used in various scenarios, from sports tracking to elderly safety monitoring devices. There are several wrist-wearable devices which enable environmental location, using the aforementioned sensor types. Determining the location could also be possible via an internet connection and wireless communication via 3G or LTE, but such Cellular Triangulation scheme techniques are not adapted for wrist-wearable devices since a telecommunication receiver is not present.

Even though it is a frequently used sensor, some challenges still arise with it, mainly accuracy as well as indoor GPS positioning problems and non-response challenges. According to GPS itself, the GPS-enabled smartphones are typically accurate in the range of approximately 5 m, since their accuracy depends on several factors, such as satellite geometry, signal blockage (i.e., indoor problems) and atmospheric conditions.

#### 4.3.10. Gravitational Force

The gravitational force, or G-force, is an acceleration measured in meters per second squared, which is the rate of change of the velocity of an object. The measurement is performed using electromechanical devices - accelerometers, which can be used to sense either static or dynamic forces, including gravity or vibrations and movement. These devices can typically measure acceleration on a three-pronged axis. The accelerometer contains capacitive plates, whereby some are fixed and others move internally, and as acceleration forces act these plates move in relation to each other, thus the capacitance between them changes, resulting in the acceleration determination. Piezoelectric materials are sometimes also used. Tiny crystal structures output electrical charge when placed under acceleration.

Such sensing is used in a variety of domains, most commonly in aviation [38], whereby lately also in the sporting domain, as an impact sensor in American football or in snow sports [98]. By focusing on the vibrations, the g-force sensing could be applied on a wrist-wearable, indicating or recording changes in acceleration.

### 4.4. Accuracy Issue as Future Research Direction

The accuracy of the sensors used in wrist-wearable devices is one of the main issues in the current commercial market. Many devices are considered to be good enough in different settings, excluding the clinical setting as the most delicate one. Studies are rarely executed on patients and especially on hospital inpatients. They largely focus on activity tracking such as in the study conducted by Case et al. [12] to evaluate the accuracy of step counts of commercially available devices. Their results indicate that measurements could be up to 20% lower compared to the observed step counts. On the contrary, Kroll et al. [6] conducted an observational study on patients in the intensive care unit to monitor the accuracy of the heart rate measurements of commercially available personal fitness trackers. Their results indicate that the heart rate measurements obtained by a personal fitness tracker were less accurate and consistent than the heart rate recorded by gold-standard measurements derived from electrocardiographic (ECG) monitoring. Performance was even worse among patients who were not in sinus rhythm. These results suggest that wrist-wearable devices will require more clinical studies conducted in a real-world setting to test the accuracy of devices and detect which groups of people are suitable for monitoring physiological parameters with commercially available wrist-wearable devices.

## 5. Intelligent Data Analysis

This section reviews the way in which different mechanisms are used to transform raw inputs into some form of new knowledge. With this, we are answering the research question SUB2-RQ1 about methods and approaches used in intelligent data analysis with the data obtained by the sensors on wrist-wearable devices.

The devices that show some form of intelligent output are categorized as Level 3 devices, based on the taxonomy of wearable functionalities introduced in Figure 1. The process of intelligent data analysis can be done in various ways, from basic mathematical transformations, basic rule sets, to statistical methods and machine learning models. The latter one, machine learning, recently garnered a lot of interest within the research community and is a driving force for innovations in the IoT and wearable industry.

Machine learning is a sub-field of artificial intelligence, where a machine derives new knowledge from data. It is divided into three categories:

- Supervised learning: A machine learns to map the input to the output based on already solved mappings. If the output of the machine learning model is numerical, we call this a regression analysis. If the output is nominal, we are dealing with a classification problem.
- Unsupervised learning: A machine learns to make some assumptions about data without it being labeled. The typical problem here is clustering, where machines group similar data together in a way that data in the same cluster are as close as possible but as different as possible from data in another cluster. Other uses here are anomaly detection and association rule learning.
- Reinforcement learning: A machine learns to make a decision based on feedback. Here, the machines optimize their actions to maximize the reward given.

A thorough description of the different methods associated with machine learning categories is out of the scope of this paper, but we refer interested readers to two classical books on the topic of machine learning [99,100].

The typical use cases for supervised learning in wearable devices is calorie expenditure calculations, where regression methods are used. The classification methods are used where the user’s wearable device, its actions or its state can be classified into one of the predefined classes. Examples of these cases are sleep quality assessments, illness and distress recognition, activity recognition and stress identification. The clustering analysis is used to group similar user activities and states together, to form a user profile. The anomaly detection is usually used in stress, illness or distress recognition, as is the removal of invalid or false data.

The following is a review of the intelligent methods from wrist-wearable sensor data, which are categorized by use cases.

### 5.1. Activity Recognition

A paper by Lu et al. [40] introduces a framework for learning analytics for wearable devices, presenting an implementation of a system that collects data from devices to detect actions. They tested their system in a school setting on students in a class that wore wrist-wearable devices to collect data. All of the action detection is done in the cloud, not on the device itself, which is out of the context of our research, but the paper still provides a great framework, that can be eventually migrated to the device in the future as these devices become more powerful and as dedicated low-power and efficient AI chips are introduced. The framework includes multiple different machine learning methods, such as classification with naive Bayes, decision trees (the authors did not mention which tree building algorithm), support vector machine and artificial neural network (again, the authors did not provide the architecture or weight tuning algorithm used with this ANN). The authors also introduced basic feature extraction with correlations, covariances, mean and magnitudes of features.

Booth and Goldsmith presented a method of gesture detection with wrist-wearable devices [83]. They experimented with support vector machine classification method to classify different hand gestures from different raw and processed signals from the device. The classification model built using their method is only applicable to the person whose gestures were used in the training process of the learning phase. All of the classification model learning and the classification itself was done on a computer and not on the wrist-wearable device.

Gjoreski et al. [7] tested different approaches to recognizing daily activities and to detect falls. They used numerous classification methods: the J48 decision tree building method, the Random Forest ensemble method, naive Bayes, support vector machines, and the k-nearest neighbors method. The only sensor in the wrist-wearable devices they used was an accelerometer. This is a rare case where the classification process was done on the wrist-wearable device.

Zhang et al. [3] presented an interesting paper where they predicted the activity of eating (specifically, over-eating) among people wearing a wrist-wearable measuring device. Their base classification method was Random forest, which used data from an accelerometer, gyroscope, pitch, and role of the device.

Sarcevic et al. [82] presented a system which recognizes a person’s arm and body movements using wrist-wearable sensors. The inputs were accelerometer, gyroscope, and magnetometer. All of the processing was done on a remote computer, not on the device. The experiment included many different machine learning approaches, from dimension reduction with LDA, to traditional classification methods such as k-nearest neighbors, support vector machines, neural networks like the method of multilayer perceptron, and nearest centroid classifier.

The paper by Bai et al [56] presents a wristband system (Microsoft Band was used in the experiment) that identifies player activity in a basketball game. In the experiment with the wrist-wearable device, they used the following classification algorithms: Random forest, naive Bayes, decision trees (construction algorithm was not defined), support vector machines and k-nearest neighbors. Basketball players also had a smartphone in their pocket that was connected to the Microsoft Band, and data were analyzed after the experiment had taken place, so there was no processing done on the wrist-wearable device itself. The inputs were only processed accelerometer data.

A team led by Vepakomma [101] presented a framework for recognizing fine-grained and complex human activities, called A-Wristocracy. They used deep learning on wrist-wearable sensors to recognize one of the 22 predefined activities.

Similarly the team by Boateng [102] developed an activity monitoring application called ActivityAware that can determine the activity levels by users using a support vector machine classifier.

### 5.2. Distress / Illness Recognition

Zangroniz et al. [5] presented a machine learning system for the classification of calm and distressed states of subjects based on an unobtrusive wrist-wearable device. Heavy signal processing is involved to transform raw inputs for electrodermal activity into usable features. The classification is done using different classification methods on a remote computer and not on a wrist-wearable device. This research was built on findings from Setz et al. [103] where they introduced a method of discriminating (classifying) people into distressed states using electrodermal activity measured by wearable devices.

Another paper by Kurniawan and colleagues [104] uses the same approach to classify individuals in a distressed or calm state using an EDA signal with k-means, Gaussian mixture models, decision trees and support vector machines. In addition, Guo et al. [105] used a galvanic skin response to classify people into calm and distressed states using the machine learning method k-nearest neighbors.

Sendulescu et al. [106] introduced improved distress classification based on the EDA signal and heart rate variability with support vector machines, while Sharma et al. [107] used evolutionary methods to classify distress with an EDA signal.

Salafi et al. [108] extended the above approaches with the raw signals of heart rate variability and skin temperature along with a standard EDA, where they used an SMO classifier on a remote computer.

Gjoreski et al. [44] presented a novel approach on stress detection, where stress is recognized in 2 and 20-min intervals from the signals (accelerometer, heart rate, EDA, the time between individual heartbeats, skin temperature) from wrist-wearable devices. They experimented with the following classification algorithms: J48 tree building classifier, naive Bayes, k nearest neighbors, support vector machines, bagging, boosting, Random forest and other various classification ensembles. The processing of data was done on a dedicated processing unit, be it a remote computer, smartphone or tablet.

Sharma and Gedeon [2] published a survey paper on stress recognition where among the sensors and measures they also presented some computational methods to transform raw signals into usable features and approaches on stress recognition. They presented the results of third-party research on stress recognition and recognized that most used classification methods are the following: various Bayesian classifiers, different approaches for constructing decision trees, support vector machines with various kernels, artificial neural networks with various architectures and neuron types, Markov chains and hidden Markov models, fuzzy techniques and a combination of all of the listed approaches.

Zheng et al. [1] published a paper where they make a comprehensive study on wearable devices for health informatics. The paper includes a section on how sensing works with data gathered by wearable devices. They focused on three different approaches for how to make predictions and diagnoses based on various inputs: the statistical approach, probabilistic approach, and artificial intelligence. They did not limit their research to wrist-wearable devices or on the processing on the wearable unit itself. Nevertheless, their paper presents a good overview of wearable devices in health.

Sandulescu et al. [106] published their research on stress detection with wearable sensors with sensors such as EDA, pulse plethysmograph, EDA, skin conductance activity and galvanic skin response. Their stress recognition service was done offline on a remote computer using support vector machine method. Similar research was carried out by Kurniawan et al. [104] who made a stress recognition system using speech and galvanic skin response inputs. Their research was not done with wearable devices, but participants had their speech and galvanic skin response measured in an experimental environment. They used decision tree classifiers (they did not provide information on which one), support vector machines with RBF kernel, Gaussian mixture model, and k-means classifier.

### 5.3. Sleep Quality

In a sequence of papers by a team led by de Arriba-Perez [39,41,42], a machine learning system for sleep quality classification is presented. Their system runs offline on a remote computer, not on a device, using classification methods such as Kstar to classify sleep patterns into one of the pre-existing classes based on the Pittsburgh Sleep Quality Index. The features used in the machine learning process are sleep duration, falling asleep, awake, heart-rate minimum, average skin temperature and quiz answers which are done on a remote system. The questions serve to appoint the person to one of the groups which then serves as one of the inputs into the learning system.

A paper by Alfeo et al. [51] presents a system that used machine learning techniques to determine sleep quality from accelerometer and heart rate sensors on a wrist-worn Android smartwatch. They used fuzzy clustering to determine two sleep quality classes and used the stigmergic receptive field in multilayer architecture to classify sleep patterns. They used Bayes networks, stochastic gradient descent for support vector machines, simple decision stumps, and decision tables in their experiment, which was done offline on a remote computer, not on a smartwatch.

### 5.4. Stress and Well-Being

De Arriba-Perez, et al. [41] presented a system for a day sleepiness level indicator. This is done via classification with the J48 classifier, which itself is a Java implementation of the popular C4.5 algorithm. Again, the learning process of three days and the sleepiness level calculations are all done on a remote computer. The inputs to classification systems are accelerometer data, heart rate, skin temperature and sleepiness type (determined by the questioner).

The same system by de Arriba-Perez predicts the chronotype of a person - the propensity for that person to sleep at a particular time during the 24 hour period. Again, all of the classification and predictions are done offline based on the following inputs: start bedtime and end bedtime.

In the same system, they also provide the stress level monitor. Again, they used a J48 tree inducing classification method on a remote system, which takes the following inputs: heart rate, skin temperature, and galvanic skin response.

The same research team later published a paper [42], in which they compared numerous different classification algorithms on the ability to correctly classify sleep-related patterns (sleepiness level, chronotype, sleep quality). The algorithms used were C4.5 tree-building algorithm, bagging (with no detailed information on base classifier used and the size of the ensemble), AdaBoost (again with lack of information on base classifier and the size of the ensemble), k-nearest algorithm, Random forest (no information about the size of the forest ensemble), naive Bayes, OneR one rule classifier, and ZeroR baseline classifier with only a majority rule for the class. The inputs here were similar to their previous research: accelerometer data, heart rate, and skin temperature. All of the processing was done on a remote computer.

Alberdi et al. [43] published a review paper on stress recognition approaches where they gathered research on which signals were used and with which methods to recognize stress. The authors did not limit themselves only to wearable devices to either measure or recognize inputs and stress, so the findings with this paper are not a direct match to this paper, topic-wise. Nonetheless, we still recognize the value in this paper as it still serves as a great review of stress recognition approaches, which can be used in building wrist-wearable devices with the goal of stress recognition.

Yu et al. [54] measured heart rate and movements with an accelerometer and skin temperature with a wrist smart band. They did not use any machine learning methods, but only linear regression between different inputs and concluded that some of these parameters could be used to predict health status and to recognize chronic diseases and emergencies.

### 5.5. Energy (Calorie) Expenditure

Alfeo et al. [50] presented a measuring device for the physical activity of older adults. The inputs were heart rate, wrist motion and pedometer, all of which were sensed by a wrist-wearable device. They used a differential evolution algorithm in combination with the multilayer of stigmergic receptive fields that classified individuals to one of the pre-determined activity levels.

Sugimoto et al. [22] developed a wrist-wearable calorie monitoring system that used pulse sensing and monitoring to calculate energy expenditure by estimating oxygen uptake from a correlation between heart rate and oxygen uptake.

### 5.6. Summary of Intelligent Data Analysis Research

The process of reviewing the methods of intelligent data analysis led to an answer for the SUB2-RQ1 research question. We found that there are numerous machine learning methods used to transform raw inputs into new knowledge. The machine learning methods range from support vector machines and decision trees to ensemble machine learning models and artificial neural networks. In addition, some unsupervised methods show up in the research literature, such as clustering with k-means. Most of the reviewed papers conducted research where they compared multiple types of algorithms to determine the most suitable for their case. Because of the no free lunch theorem [109], there is no use in making conclusions of the best algorithms, because not only can we not proclaim the best algorithm universally, but it is also bold and foolish to conclude the best algorithm for any particular type of data. Every result and the conclusion of every paper can only be generalized for the data used in that particular experiment. While we can still use the list of used algorithms as a guide for any future research, we should not limit ourselves to using only one algorithm based on existing research. Next, papers lack the descriptions of their limitations, be it in computational power, or usage of open source libraries, which limits the usage of the wide body of algorithms. In addition, as we have seen, there is a limited use of current state-of-the-art algorithms. While some researchers use Random Forest and other ensemble boosting methods, none of the papers cited any variation of the gradient boosting method, which has been sweeping the Kaggle competitions [110]. In addition, there is only one paper we found that used deep learning neural networks in analyzing the data from wrist-wearable devices. While we acknowledge that there are numerous shortcomings to the said gradient boosting and deep learning methods, namely computational complexity and the complex nature of hyper-parameter tuning, most researchers ignore these approaches. For these reasons, we refrained from drawing any conclusions about the best algorithm for any use case.

We also detected an issue related to the lack of on-device usage of the machine learning model. The main body of research uses off-line data analysis, where the sensing and logging are done on the device and then the data is downloaded to a remote computer with greater computational capacities, where an analysis is done.

Other than a review of algorithms, we find that the review of used inputs into machine learning algorithms could provide a valuable resource for any future research. Table 4 shows the input data that was identified for use in the particular use cases in intelligent data analysis.

## 6. Visualization of Measurements

Usually, sensors produce raw data for measurements in the realm of number presentations (also called time series of scalars [111]). These presentations are mostly direct measurements, while sometimes devices conduct primitive operations in order to improve the user’s experience. Direct measurements are measurements that are taken directly from a sensor without any special pre-processing techniques. In this group, we can count step counters or temperature meters. On the other hand, there is also a large group of sensors that produce measurements, but need some pre-processing techniques in order to produce valuable information for their users [112]. For example, a modern running watch has an integrated GPS sensor that obtains a real-time position that is expressed in latitude and longitude [113,114]. These measurements are not directly important to users, while processing them produces valuable information for users. In line with this, differences between latitudes and longitudes can output the following information: current speed, total distance, max speed.

These values are very important for athletes. Nowadays, many athletes train while using smart watches [115]. For that reason, quick feedback is desired in order to monitor progress during the whole training session [116]. In contrary, such modern wrist-wearable devices are also able to log activities during training [117]. After the training session, athletes can transfer the whole activity either onto a computer, mobile device or to any web applications that are intended for a post-hoc analysis of sport activity.

Due to the desire for a quick response and the hardware limitations of wrist-wearable devices, more powerful visualizations are mostly done post-hoc (after the activities or even after a particular number of conducted activities). In fact, people are visual beings that deal much more easily with a visual representation than a numerical representation. Basically, a visual representation tells us a much greater story than purely numbers. For that reason, researchers have developed some sophisticated visualization techniques that can easily visualize sporting activities on the one hand, while on the other hand it can produce effective comprehension. There are many visualization methods that are suitable for visualizing measurements that are produced by sensors of wrist-wearable devices. Our study revealed that researchers used the following methods for visualization, i.e., 3D visualization, dashboards, line plots, glyph-based visualizations, physical visualizations, Poincaré plots, trajectory plots and vector plots (see Table 5).

Fens and Funk [118] visualized measured physiological parameters using a 3D visualization method that results in a three-dimensional data landscape. This visualization is also interactive, which allows the users to pan, zoom and rotate in order to change views on data. Fens and Funk [118] also visualized health data using the dashboard [119] style approach that consists of line graphs [111] as well as interactive elements to zoom in and toggle the visibility of individual data streams [118]. On the other hand, physical visualizations is also an interesting approach, in which data is moved to the physical world. This can improve the users’ efficiency with information retrieval tasks [118,120]. In line with this, glyph-based visualization is also one example of effective information visualization of sensor data produced by wearable wrist-band devices. According to Ward [121], glyphs are graphical entities [122] that convey one or more data values via attributes such as shape, size, color, and position, and thus improve the user’s experience. Some examples of glyph-based visualizations of sensor data are presented in papers [49,123]. A Poincaré plot is a graphical representation tool for time series analysis that visualises a scatter of point clouds and the relationship between the points [124,125]. In contrast, a trajectory plot is appropriate for the analysis of positional data [111]. Software also exists for data visualization called TinkerPlots [126] which is also able to visualize data produced by wearable devices [127].

Currently, there is also a lack of guidelines about the design of visualization [128] that is appropriate for small displays and for use in real scenarios. Researchers are working intensively on solving this problem. Interestingly, one paper [128] reported results of perceptual studies with smartwatches under realistic conditions. Hence, we can expect that even more guidelines will be released in the future.

### Examples of Sport Activity Visualization

According to the literature review, most devices enable the post-hoc visualization of measurements. Especially, producers of wrist-wearable devices that are intended for sports also provide web applications that allow athletes to upload activities for future analysis. The visualization of activities is an essential part of many functionalities that are developed within web applications. In line with this, the visualization of activities helps athletes gain more insight into the workout due to the many graphs and charts. Some of these applications are: Garmin Connect (https://connect.garmin.com), Strava (https://www.strava.com/), Movescount (http://www.movescount.com/), Polar (https://flow.polar.com/), Endomondo (https://www.endomondo.com/), and Runtastic (https://www.runtastic.com/). Some of these applications were released with mobile applications for tracking sport activities [117,129].

**Figure 2:**
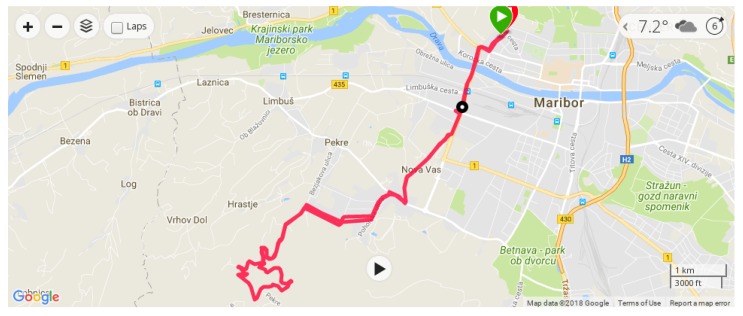
Trajectory plot (full map) of a short mountain bike ride that was completed in Maribor (Slovenia). The data were recorded by a Garmin Vivo Active HR watch that allows one to measure one’s heart rate directly on the wrist.

**Figure 3:**
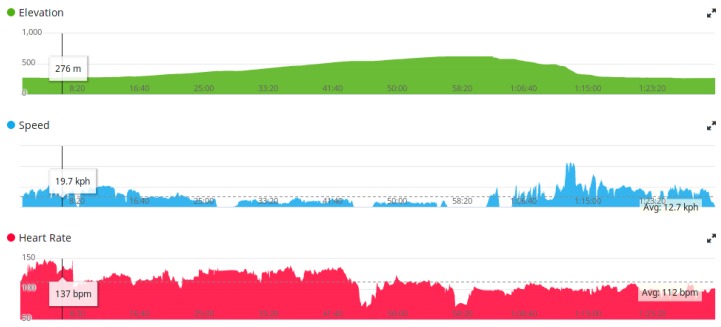
Figure presents visualized graphs of the three main parameters of workout (Figure 2), i.e., elevation, speed, and heart rate according to time.

Figure 2 and Figure 3 present one mountain bike ride that was completed by a semi-professional cyclist and uploaded onto Garmin Connect. The conducted activity was actually an easy winter ride. Figure 2 presents an exact map of the ride, while Figure 3 presents graphs of the main parameters within the activity, i.e., elevation, speed, and heart rate according to time. These graphs help athletes and trainers make an easy analysis of the activity.

**Figure 4:**
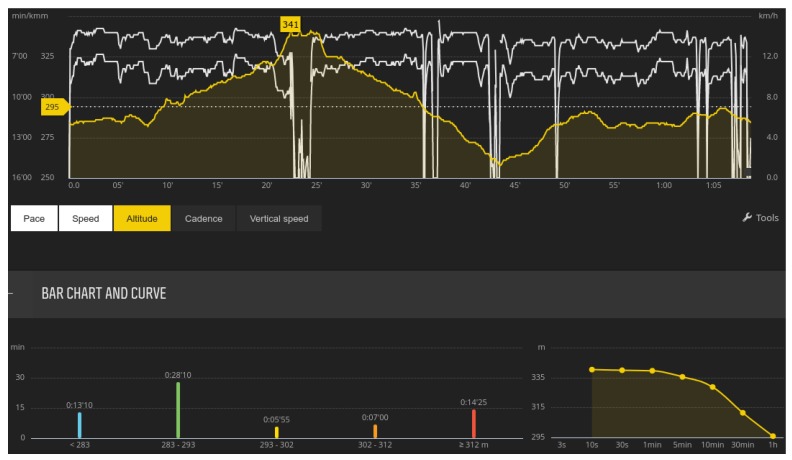
Visualization of running activity on the Movescount platform.

From Figure 2 and Figure 3, we can easily infer that an athlete had a really low pace along with many stops during the ride. The heart rate was higher in the first part of the training session, but fell in the second part. It is also supported by the elevation. In the first part of the activity, the athlete was riding uphill, while the second part of the race course was mostly downhill. Figure 4 presents a visualization of running activity that was uploaded onto the Movescount platform.

**Figure 5:**
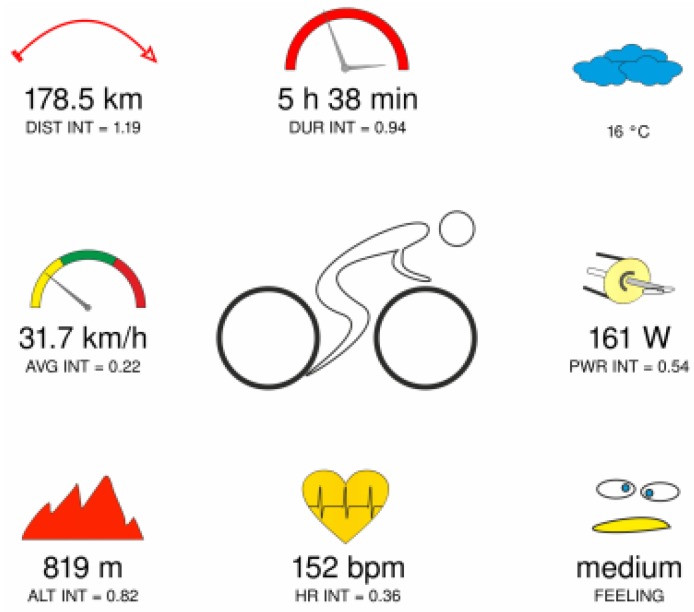
Figure shows the main basic parameters of sport training. The main basic parameters that are part of every cycling training are: total duration, total distance, temperature, average speed, altitude, power-meter values and average heart rate. Note: The Figure is reproduced with permission from [123].

An example of a glyph-based visualization method that constitutes an example of visualized cycling activity, more precisely mileage training, is presented in Figure 5. As we can deduce from the figure, entities such as colors and graphical elements play a big role in presenting an activity, while it is even supported by the number presentation in order to enrich the user experience.

## 7. Discussion

In this paper, we focused on analyzing wrist-wearable devices, reviewing the sensors used in those devices as well as methods for the intelligent analysis of data obtained by those sensors. We also reviewed methods used for the visualization of measured parameters and analyzed popular commercial wrist-wearable devices. The analysis revealed that several wrist-wearable devices on the market include one or several sensors, chosen according to the specific domain of interest. The most common cases are those of health monitoring and sports tracking, while more specific ones were also identified. Additionally, we also identified several prototype wrist-wearable devices, which use a specific set of sensors or a specific new type of a sensor, commonly not present in devices sold on the market. We also identified sensors type never presented as part of a wrist-wearable device, but could be, due to their structure and architecture.

The performed literature review concluded that, so far, a taxonomy of wrist-wearable devices was not proposed. Our first research question RQ1 and its sub-questions led us to detect the core elements for a taxonomy of wrist-wearable devices. Therefore, we synthesized a taxonomy of sensors, functionalities and methods used in those devices. To answer the first and the second sub-questions SUB1-RQ1, we reviewed the sensors integrated into non-invasive wrist-wearable devices used to measure human physiological and activity parameters, as well as environmental parameters. These devices are used to measure in a non-invasive manner, the following physiological parameters: heart rate, body temperature, blood pressure, blood oxygen saturation, blood sugar, blood volume pulse, electrodermal activity, and electrolytes. Activity sensing is done through motions, gestures, acceleration, strain, and proximity detection signals. Finally, environmental parameters, captured by wrist-wearable devices, are air temperature, altitude, light, noise, atmospheric pressure, humidity, gas, location, and g-force. The review revealed that the heart rate is the most measured physiological parameter by commercial wrist-wearable non-invasive devices. Heart rate is mostly measured using a PPG sensor, which is mostly based on finger measurements sites in commercial solutions [72]. However, there are many applications and research studies that address different issues related to placing PPG into a wrist-wearable devices and obtaining accurate measurements. Due to open issues (e.g., motion artefacts influencing the accuracy of measurements), these devices are usually not used in clinical settings. The review also revealed that the blood oxygen saturation and blood sugar are measured in some researches and prototypes, but are still not integrated into the commercially available devices. Regarding environmental parameters, some prototypes also mention the possibility of using a g-force sensor on a wrist-wearable device [38], but such devices are not currently in commercial use. To summarize the findings, despite the large number of sensors that were found in the literature, the most common sensors used in wrist-wearable devices are the heart rate sensor and motion sensors, such as accelerometer and location sensors, while others are included only sporadically [41]. When combining sensors into a device it is important to select sensors that are complementary and that can cover the recognition of a larger range of activities.

In finding the answers to research question SUB2-RQ1, we made a thorough review of intelligent systems that work on, or with, wrist-wearable devices. The main takeaway from it is that there is a general lack of onboard intelligent systems on the device itself. The majority of research where data from sensors on wrist-wearable devices were used in data analysis process, was done offline—after the data gathering process. While this approach still provides a valuable insight into the usability of intelligent methods in such scenarios, this is not applicable to production-ready devices. The most common cases of intelligent analysis are the following: (1) gathering data from wrist-wearable devices; (2) using machine learning methods on a remote, offline computer to gather insights about the data and build a machine learning model; (3) evaluating the model and comparing it to competing approaches; and (4) using prebuilt machine learning models on the device. The second step is the most expansive, computationally-wise, and therefore demands specialized hardware, which is still not present in the appropriate form factor for wrist-wearable devices. In addition, there is a lack of usage in the latest state-of-the-art machine learning methods, namely deep learning neural networks, gradient boosting and other ensemble methods. While there are research papers that have experimented with numerous machine learning methods, we cannot reach a conclusion on the best methods for usage in any use cases, because of a lack of descriptions on the limitations of the device, experiment or research in general in those papers and also because of the no free lunch theorem, stating that there is no universally best algorithm and that the results can only be generalized for the data used in the experiments. The last, fourth step is the one that bridges the gap from the research findings to the actual integration into a device. This step is still avoided in the research community and is mainly left to the industry. The use of prebuilt machine learning models is still the most computationally expensive process, but not nearly as expensive as the building process of these models, which is why we already see them used in smartphones. However, the step of using the models on the actual devices for real-time intelligent analysis presents the last and the hardest mile in the research process and is thus overlooked far too often. Otherwise, there is an eager research community that develops and integrates the latest state-of-the-art machine learning methods in the analysis of data in all tracking devices. We can expect this trend of implementations of latest machine learning methods in the wrist-wearable devices in the future, with even more adoption and applications as hardware advances and machine-learning algorithms are optimized for low-powered and specialized processing units.

To address the research question SUB3-RQ1, we made a review of different visualization techniques used in wrist-wearable devices. Similarly, as with the state-of-the-art machine learning methods, state-of-the-art visualization techniques are also computationally expensive. There is still much room for improvement in the visualization of parameters as well as activity measurements that are produced by various sensors. Therefore, they are inappropriate for direct usage on devices/products. Hence, visualization is mostly conducted offline after monitoring/tracking, while only primitive visualization is performed directly on the device. While visualizations are used to present the raw outputs of sensors, such as heart rate, blood pressure and other parameters, there is a lack of interplay of visualization techniques that show the results of the intelligent data analysis.

As we saw in Section 3, where we took a deeper look into existing devices, some of the intelligent methods are already implemented on the devices, on the companion devices (such as a mobile phone) or in the cloud. Especially in the latter case, all of the findings from this research can be applied but carries the negative consequence that the device has to always be connected to the Internet always to take advantage of the computing power of the dedicated powerful machines in the cloud. In the case of onboard intelligent analysis, this area still lacks the application of bleeding edge findings from research as there are still some serious hardware limitations for such devices, be it in computing power or battery capacity.

Our research revealed that the border between commercial products and research prototypes is very narrow (RQ2). Commercial products mostly use older, low cost standardized and well-established sensors for monitoring/tracking health/activities. On the other hand, research prototypes are proposing new sensors that involve new functionalities which have still not matured enough for inclusion in commercial products. For example, a sensor for the non-invasive measuring of blood sugar has recently been proposed as a proof of concept [81].

## 8. Conclusions

We have reviewed recent advances in the development of non-invasive wrist-wearable devices. The aim of the review was to analyze the current trends for sensors used in wrist-wearable devices, methods for visualization and intelligent data analysis, as well as to compare popular commercial wrist-wearable devices. Because of a lack of a clearly defined taxonomy, one of the results of the review is also the taxonomy of sensors, functionalities and methods used in wrist-wearable devices.

The onboard device-intelligent analysis of the data will become easier in the future as the trend of specialized machine learning chips becomes more prevalent. An experiment with Google’s Tensor processing unit (TPU) [130], which specializes in operations needed for learning and using artificial neural networks, shows great improvements for the usage of production neural network applications. While Google’s TPU is not optimized for hardware size and as such is not usable in mobile devices, similar future mobile chip development can be expected.

Most of the mobile phone chip makers already include a graphical processing unit to their chip sets, but projects such as ARM’s Trillium (https://developer.arm.com/products/processors/machine-learning/arm-ml-processor), Apple’s A11 chip with onboard neural network engine (https://developer.apple.com/machine-learning/) and Samsung’s new Exynos 9810 also with neural neural network capabilities (https://news.samsung.com/global/samsung-optimizes-premium-exynos-9-series-9810-for-ai-applications-and-richer-multimedia-content), show a trend for the future of mobile processing chips. As these chips become cheaper and more accessible, we can expect more AI powered wrist-wearable devices. These AI tailored chips are similar to GPUs where they specialize in matrix algebra operations, are more tolerant to reduced computational precision and thus require fewer transistors per operation. Fewer transistors means less battery is consumed for any particular operation.

Advances in hardware will also drastically open a new window for more sophisticated visualizations. Even though most visualization is currently conducted by web applications or smartphones that are connected with devices, up and coming devices will allow for the real-time visualization of measurements directly on the device itself. On the one hand, this would improve the user’s experience, while, on the other hand, it should encourage more research in the visualization field.

Open room for improvement is also present in the field of sensor research. Available sensors for the non-invasive measurement of physiological parameters are capturing small signals with a lot of noise. The noise has to be reduced using specific algorithms that filter the raw signal and amplify only the desired part of the signal. Future developments should improve existing sensors, accustom the existing sensor for wearable device use, and also contribute to the creation of new ones, which should eliminate the necessity for signal processing before the usage of data. Another issue which deserves attention is the homogenization of data, where every device vendor stores data in their own style instead of in accordance with a common convention. Papers published by de Arriba-Pérez et al. [41,131] are valuable resources addressing this issue. Furthermore, during the review, it was noted that several prototypes exist which experiment with the inclusion of additional specific sensors, such as g-force, saturation, and others, onto a wrist-wearable device. In the future, these prototypes will become commercially accessible wearable devices, thus bringing additional data into the data gathering process.

## Figures and Tables

**Table 1:** Market share according to IDC Research Inc. [66].

Vendor	2017 Market Share
Apple	15.3%
Xiaomi	13.6%
Fitbit	13.3%
Garmin	5.4%
Fossil	4.3%
Others	48.1%

**Table 2:** Commercial devices in a nutshell.

	Commercial Devices
Key features	Sleep tracking, Heart rate monitoring, Step tracking, Water sports support, Stroke detection, Coaching support, Breathing support
Sensors	3-axis accelerometer, Vibration motor, motion sensing system, Gyroscope, Magnetometer, GPS
IT support	Smartphone notifications, Support for various mobile OS including iOS and Android, Web applications

**Table 3:** The overview of sensors used in physiological, activity and environmental sensing.

		Physiological Sensing						Activity Sensing			Environmental Sensing								
		**Heart Rate**	**Body Temperature**	**Blood Pressure**	**Blood Oxygen Saturation**	**Blood Sugar**	**Blood Volume Pulse**	**Motion and Gestures**	**Body Acceleration**	**Proximity Detection**	**Air Temperature**	**Altitude**	**Light**	**Sound/Noise**	**Atmospheric Pressure**	**Humidity**	**Chemical/Gas**	**Location**	**Gravitational Force**
SENSORS	Photoconductivity	X	X	X		X							X					
	Multi-channel WPPG	X																	
	Infrared	X	X							X									
	Termistor		X																
	Thermoelectric effects		X								X								
	Pressure/Strain			X				X				X		X	X			X	
	Optical (photodetector)		X		X								X						
	Electrochemical					X													
	Accelerometer	X						X	X									X	X
	Gyroscope							X										X	
	Magnetometer							X		X								X	
	RGB–D							X											
	Surface electromyography							X											
	Ultrasonic									X									
	Radio Frequency									X									
	Magnetic Field									X									
	Radar									X									
	Sonar									X									
	GPS									X		X						X	
	Piezoelectric-based			X				X							X	X	X		

**Table 4:** Type data used in intelligent data analysis.

		Activity Recognition	Distress/Illness Recognition	Sleep Quality	Stress and Well-Being	Energy (Calorie) Expenditure
INPUT DATA	Heart Rate	X	X	X	X	X
	Skin Temperature		X	X	X	
	Blood Pressure		X			
	Motion and gestures	X	X	X	X	X
	Body Acceleration	X	X	X	X	X
	Proximity detection	X				
	Sound/Noise			X		
	Electrodermal Activity (EDA)		X			
	Galvanic skin response		X		X	
	Gravitational force	X				

**Table 5:** Visualization methods that are used for the visualization of measurements produced by wearables.

Method	Reference	Domain
3D visualization	[118]	Health
Dashboard	[118]	Health
Line plot	[111]	Sport
Glyph-based visualization	[49,123]	Sport
Physical visualizations	[118]	Health
Poincaré plot	[124]	Health
Trajectory plot	[111]	Health
Vector plot	[111]	Sport
